# Supplementary material for: Association between genetically proxied PCSK9 inhibition and prostate cancer risk: A Mendelian randomisation study
Source: PLoS Med. 2023 Jan 3;20(1):e1003988. doi: 10.1371/journal.pmed.1003988 (PMC9810198; doi:10.1371/journal.pmed.1003988)
Supplement: S1 Supplementary Note — (DOCX) [file pmed.1003988.s002.docx]

**Supplementary Note 1. Data sources for secondary analyses.**

In secondary analyses, *cis*-acting protein quantitative trait loci (*cis*-pQTL) for PCSK9 were used as the genetic instruments for circulating PCSK9 protein levels. PCSK9 *cis*-pQTLs were extracted from a publicly available GWAS on plasma levels of PCSK9 measured in 35,559 Icelanders [1] and clumped using a window of 100kbs around the *PCSK9* encoding region based on P<5×10^-8^ & r^2^<0.1 with the same reference panel as above. All LD clumping was performed using PLINK (v1.9). A similar protocol was applied to select instruments for *PCSK9* expression derived from liver tissue (N=208) using data from the latest release (v8) of the Genotype-Tissue Expression (GTEx) project [2]. As no liver-tissue PCSK9 *cis*-expression quantitative trait loci (cis-eQTL) reached the P-value threshold for MR analyses (P<5×10^-8^), the top eQTL (rs553741) which provided the strongest statistical evidence (P=6.02×10^-8^) and F-statistic (F=26.9) was used to derive the Wald ratio estimate for MR analyses on prostate cancer outcomes. In addition, a validation analysis was performed using conditionally independent PCSK9 *cis-*pQTLs identified by Ferkingstad *et al.* [1].

In addition, genome-wide significant variants associated with Lp(a) levels (P<5×10^-8^ & r^2^<0.001) were identified from the Neale lab UK Biobank GWAS on male participants (n= 167,020) in order to replicate previously published findings on the association between Lp(a) and prostate cancer risk [3]. There were 15 genetic variants from across the genome used as instrumental variable for Lp(a). One of these variants, rs73596816, is located in the *LPA* gene and explains more than 70% of variations in Lp(a) levels (F=4590.0).

1. Ferkingstad E, Sulem P, Atlason BA, Sveinbjornsson G, Magnusson MI, Styrmisdottir EL, et al. Large-scale integration of the plasma proteome with genetics and disease. Nat Genet. 2021;53(12):1712-21. Epub 2021/12/04. doi: 10.1038/s41588-021-00978-w. PubMed PMID: 34857953.

2. The GTEx Consortium. The GTEx Consortium atlas of genetic regulatory effects across human tissues. Science. 2020;369(6509):1318-30. Epub 2020/09/12. doi: 10.1126/science.aaz1776. PubMed PMID: 32913098; PubMed Central PMCID: PMCPMC7737656.

3. Ioannidou A, Watts EL, Perez-Cornago A, Platz EA, Mills IG, Key TJ, et al. The relationship between lipoprotein A and other lipids with prostate cancer risk: A multivariable Mendelian randomisation study. PLoS Med. 2022;19(1):e1003859. Epub 2022/01/28. doi: 10.1371/journal.pmed.1003859. PubMed PMID: 35085228.
