# Supplementary figures and images for: Association between genetically proxied PCSK9 inhibition and prostate cancer risk: A Mendelian randomisation study
Source: PLoS Med. 2023 Jan 3;20(1):e1003988. doi: 10.1371/journal.pmed.1003988 (PMC9810198; doi:10.1371/journal.pmed.1003988)

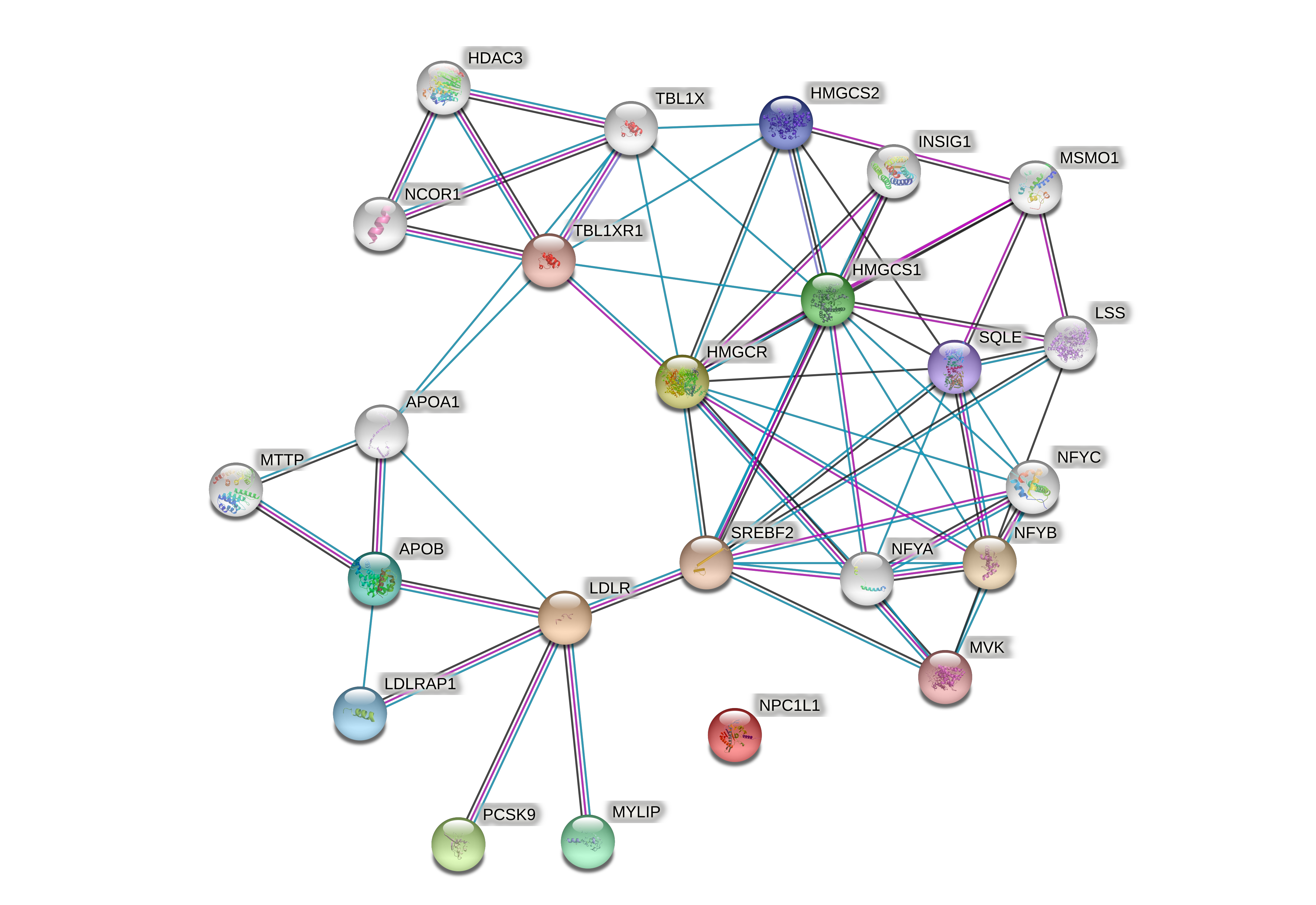

Supplement: S1 Fig — (TIFF) [file pmed.1003988.s018.tiff]

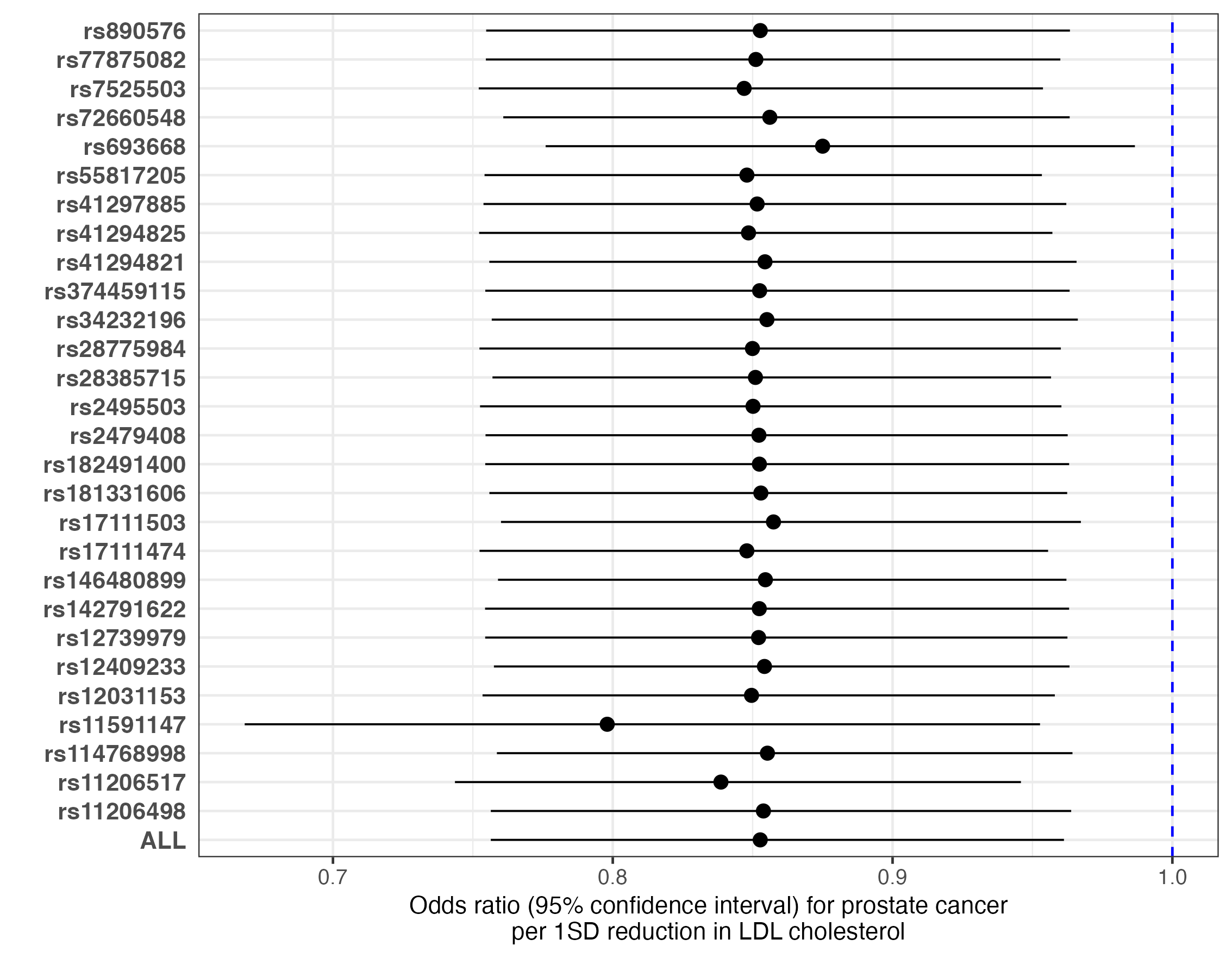

Supplement: S2 Fig — SD, standard deviation. (TIFF) [file pmed.1003988.s019.tiff]

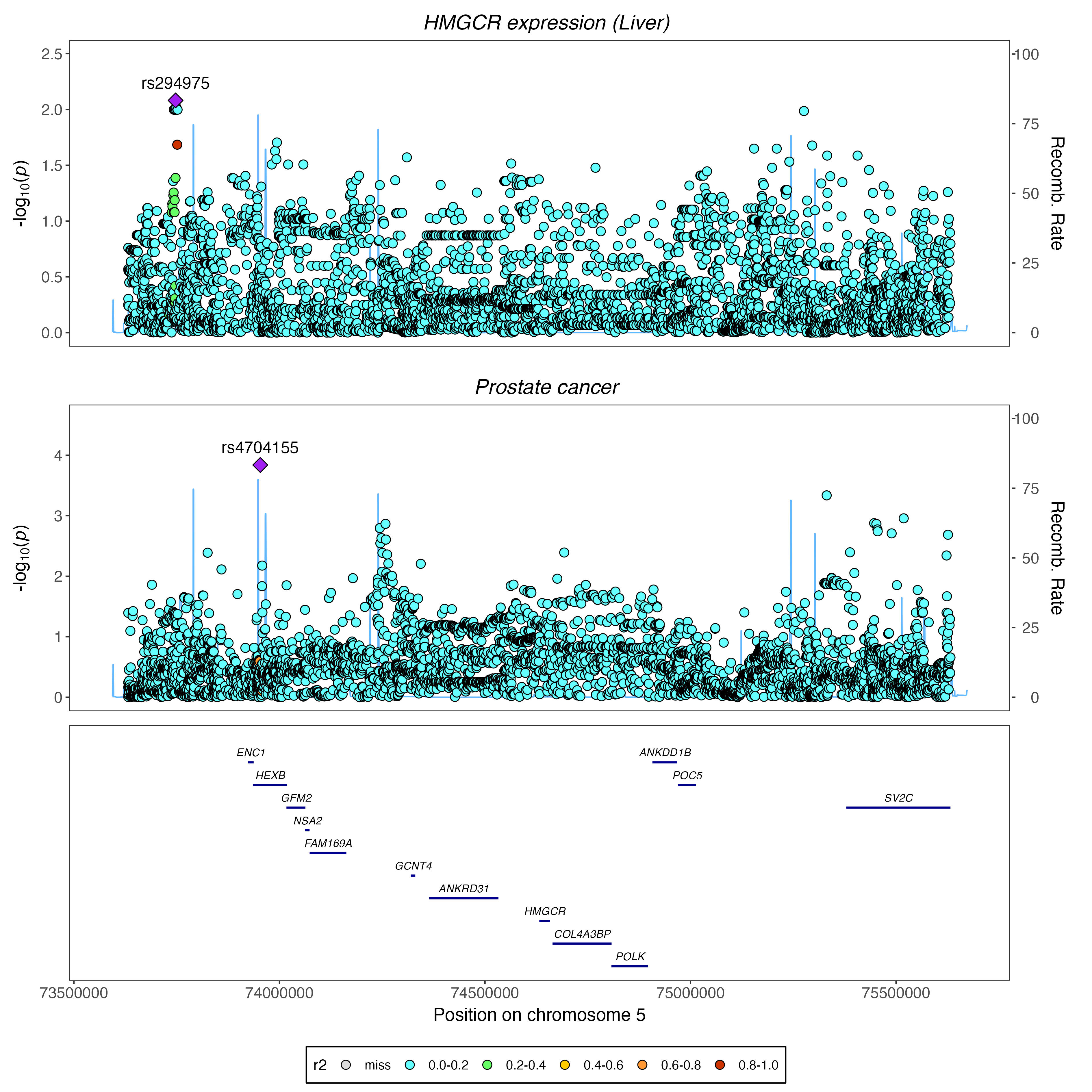

Supplement: S3 Fig — (TIFF) [file pmed.1003988.s020.tiff]

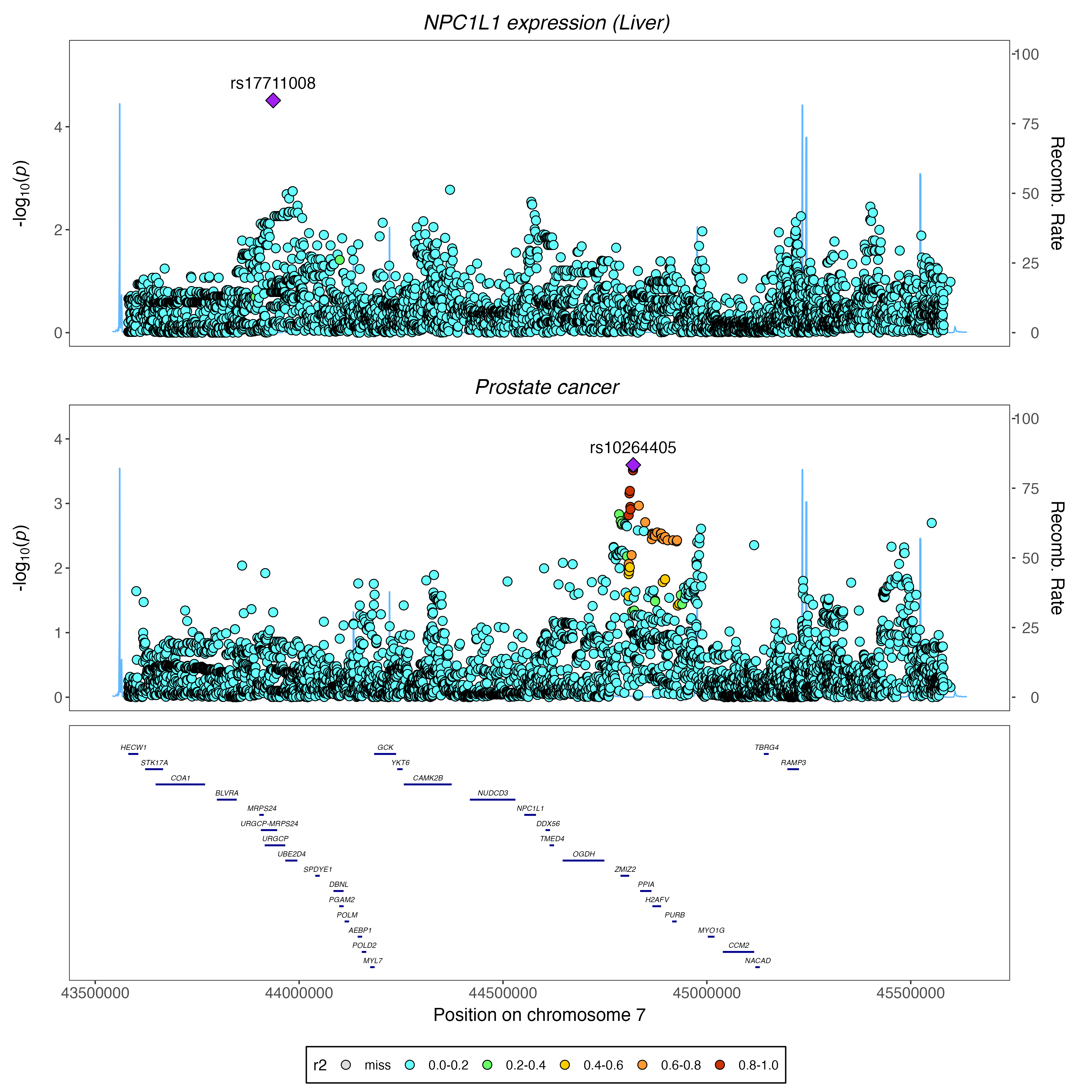

Supplement: S4 Fig — (TIFF) [file pmed.1003988.s021.tiff]

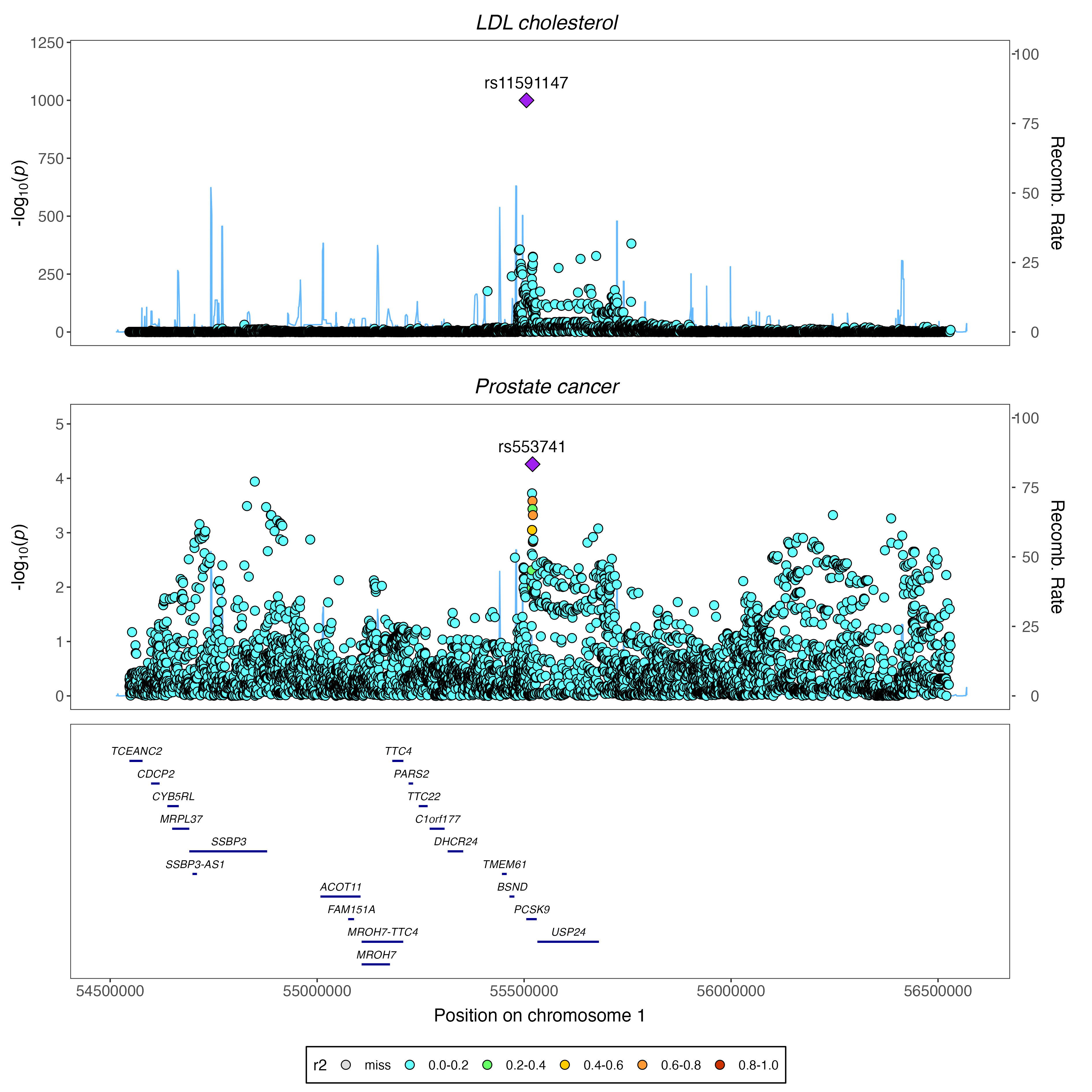

Supplement: S5 Fig — (TIFF) [file pmed.1003988.s022.tiff]

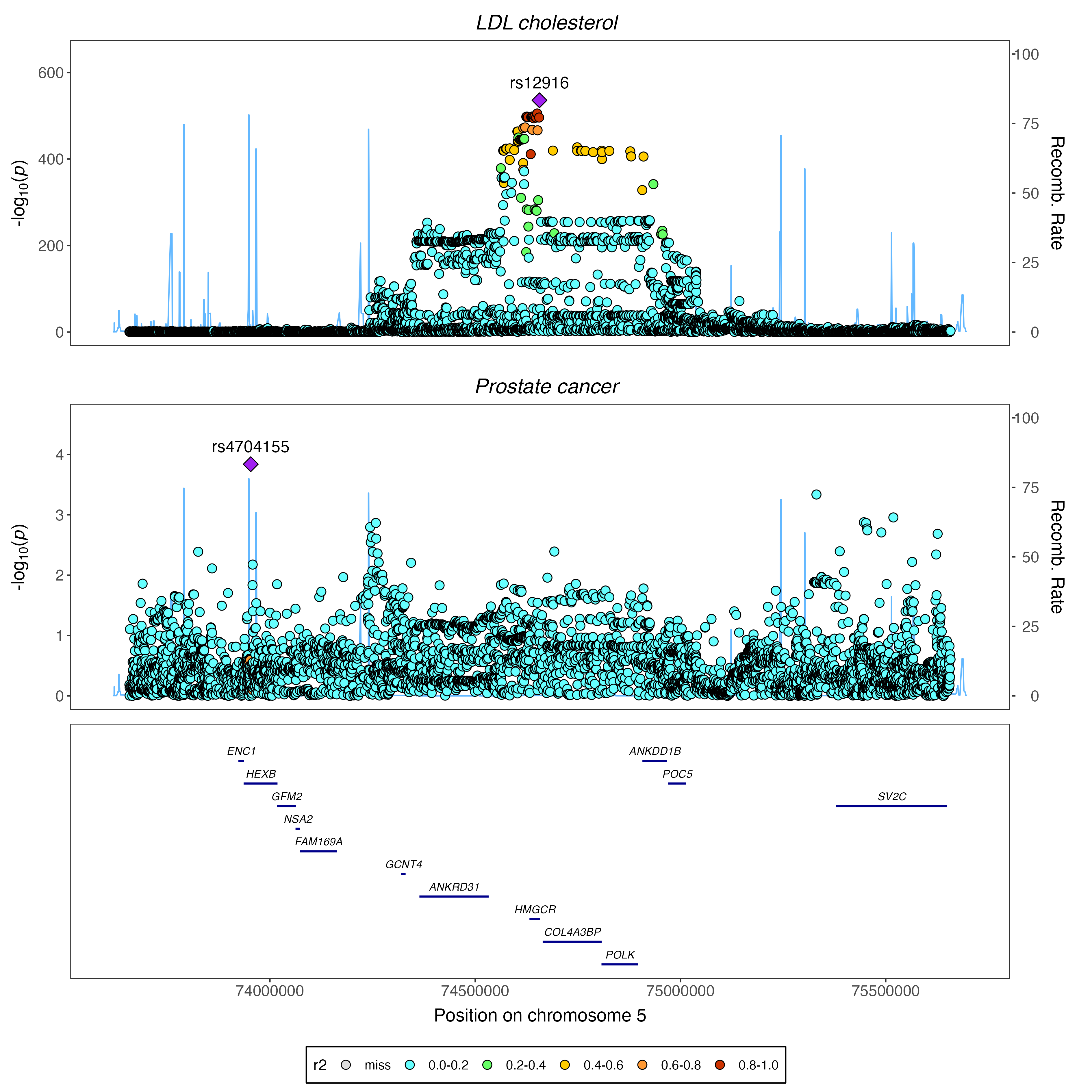

Supplement: S6 Fig — (TIFF) [file pmed.1003988.s023.tiff]

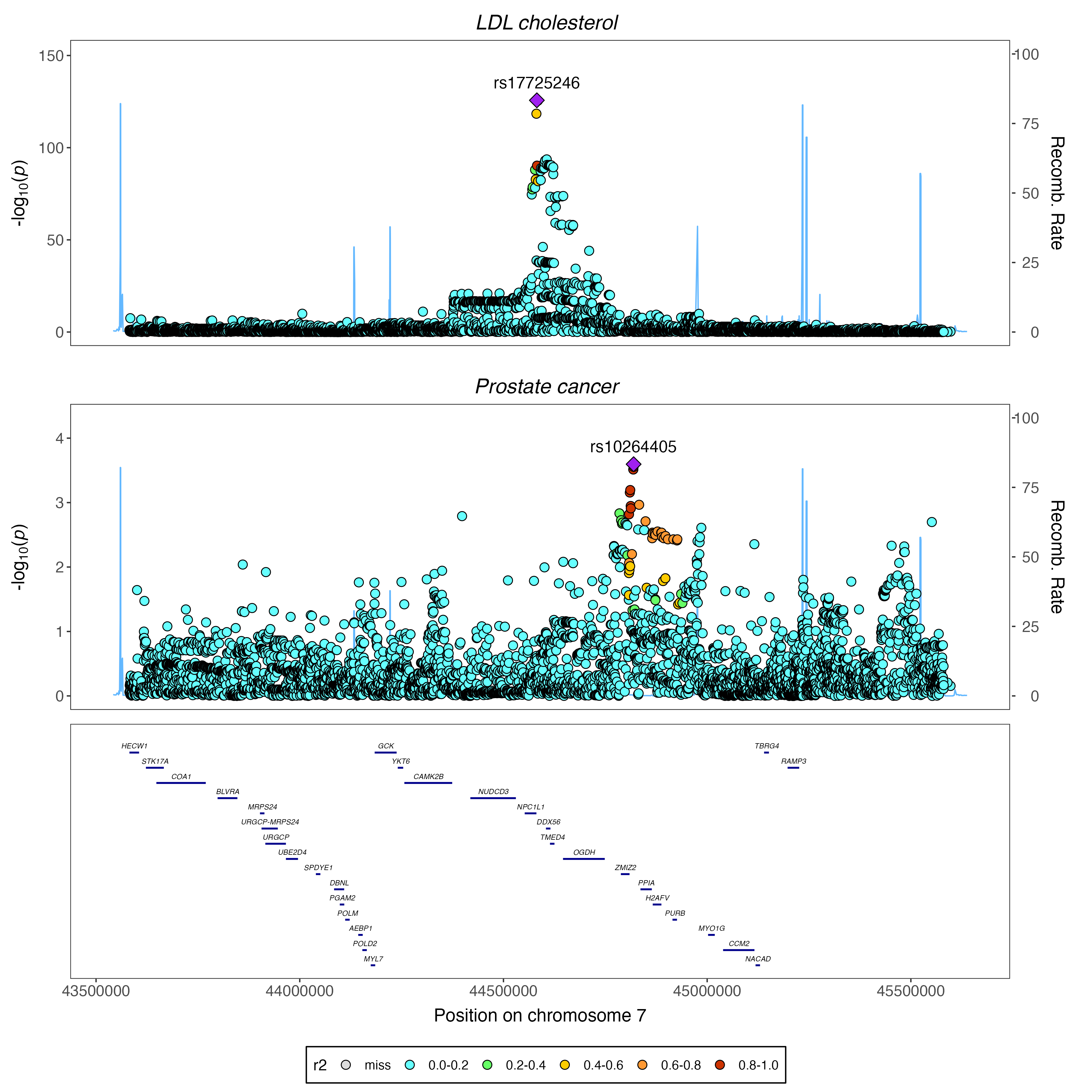

Supplement: S7 Fig — (TIFF) [file pmed.1003988.s024.tiff]
