# Supplementary material for: Association between genetically proxied PCSK9 inhibition and prostate cancer risk: A Mendelian randomisation study
Source: PLoS Med. 2023 Jan 3;20(1):e1003988. doi: 10.1371/journal.pmed.1003988 (PMC9810198; doi:10.1371/journal.pmed.1003988)
Supplement: Attachment — Submitted filename: pcak9_prostate_cancer_Response to reviewers comments_20Sep.docx [file pmed.1003988.s026.docx]

**Manuscript ID: PMEDICINE-D-22-01177R2**

**Response to Reviews’ Comments**

Dear PLOS Medicine editorial team and reviewers,

Thank you for giving us the opportunity to submit a revised draft of the manuscript “Association between genetically proxied PCSK9 inhibition and prostate cancer risk: a Mendelian randomization study” for publication in PLOS Medicine. We would also like to thank the editor and reviewers for their time and insightful comments which contributed greatly to the improvement of the manuscript.

In summary, we have made several major changes to the manuscript to incorporate suggestions from these comments:

1. In the primary analysis, we have now updated our genetic instruments for LDL-c lowering drug targets and overall LDL-c using results from the latest GLGC GWAS of lipids by Graham et al.
2. We have added replication analyses using male stratified GWAS summary statistics for lipoprotein (a) and body mass index when estimating their associations with drug targets and prostate cancer risk in MR.
3. Additional locus zoom plots visualising findings, colocalization analyses, genetic correlation analyses and how our analyses build upon previous work have all been added to the manuscript as described in detail below.

We can confirm that this manuscript has not been published and is not under consideration for publication elsewhere. For the purpose of Open Access, the author has applied a CC BY public copyright licence to any Author Accepted Manuscript version arising from this submission.

We look forward to hearing from you in due time regarding our submission and to respond to any further questions and comments you may have.

Yours faithfully,

Si Fang

on behalf of authors

**Comments from the Academic Editor:**

1) Genetic variants used to construct the instruments were derived from a paper published in 2013. It might be worthwhile to search for more recent papers to see if additional variants have been identified for these targets:

Author response: Thank you for this suggestion. We have replaced the source of genetic instruments for LDL-c and lipid lowering drug targets with the latest Graham et al. GLGC GWAS on LDL-c published in December 2021 (European ancestry including UK Biobank, N= 1,320,016). There are now more variants eligible for our instruments selection criteria which are also present in the PRACTICAL consortium prostate cancer GWAS, with 28 for PCSK9, 4 for NPC1L1, 13 for HMGCR, 36 for LDLR (*P*<5x10^-8^, r^2^<0.1, located within +/-100kb around the protein encoding genes) and a total of 424 for LDL-c (*P*<5x10^-8^, r^2^<0.001). Results from the updated MR analyses are consistent with the previous results. Relevant information has been updated in the Methods (page 9) and Results (page 15) sections.

2) The authors may want to perform a MRA to directly assess the association of Lp(a) with prostate cancer risk;

Author response: The associations between Lp(a) and prostate cancer risk has been extensively investigated in a recent publication by Ioannidou et al. (2022) in PLOS Medicine using both univariable and multivariable Mendelian randomization (MR) as well as multiple sensitivity analyses. Ioannidou et al. identified a strong association between the genetically predicted Lp(a) levels and overall prostate cancer risk in an univariable setting using weighted median model, as well as in multivariable MR using the inverse variance weighted model adjusting for HDL, LDL and TG. To replicate part of their analyses which is relevant to our study, we conducted univariable MR analysis looking at the association between genetically proxied Lp(a) and PrCa risk, as well as a multivariable Mendelian randomization analysis looking at the association between genetically predicted Lp(a) levels (using variants from the Neale lab male-stratified GWAS on inverse rank normalised Lp(a) levels) and prostate cancer risk adjusting for the levels of LDL-c.

Relevant information has been added to Methods (page 14) and Results (page 20).

3) it would be helpful to mention in the abstract that the association with PCSK9 is not mediated through reducing LDL-C;

Author response: This has been added to the Conclusions in the abstract (page 4):

“Our study supports a strong association between genetically proxied inhibition of PCSK9 and a lower risk of total and early-onset PrCa, potentially through an alternative mechanism other than the on-target effect on LDL-c.”

4) It is interesting to see the association between HMGCR and testosterone, which is a major risk factor for prostate cancer. However, no association was identified between HMGCR and prostate cancer risk. The authors may want to discuss this finding.

Author response: Thanks for the suggestion. This null association between HMGCR and prostate cancer risk identified in the drug target MR analysis is also supported by colocalization evidence (see locus zoom plots S1 Fig, S4 Fig and a *coloc* H4<0.005).

Additional discussion on HMGCR has been added (page 22):

“In addition, despite the odds ratio from MR analysis suggest there is potentially some evidence for associations between genetically proxied inhibition of HMGCR or NPC1L1 and prostate cancer, the results are not statistically significant with 95% CIs included 1. The null associations are supported by colocalization evidence at both genetic loci. This is particularly interesting given that genetically proxied HMGCR is associated with BMI and testosterone, two well established risk factors for prostate cancer. This could be caused by the lack of power of the prostate cancer GWAS at these two loci, given that the genetic variants associated with prostate cancer all have a P-value of higher than 0.001 at both gene regions. This should be further investigated in the future when a larger GWAS meta-analysis on prostate cancer is available.”

**Requests from the editors:**

Please revise your title according to PLOS Medicine's style. Your title must be nondeclarative. It should begin with main concept if possible. For example: "Association between genetically proxied PCSK9 inhibition and prostate cancer risk: a Mendelian randomization study", or similar.

Author response: The title has been revised to "Association between genetically proxied PCSK9 inhibition and prostate cancer risk: a Mendelian randomization study" as requested.

Please add this statement to the manuscript's Competing Interests: "GDS is an Academic Editor on PLOS Medicine's editorial board."

Author response: This statement has been added to the competing interests on page 2 as requested.

Abstract Methods and Findings:

* Please ensure that all numbers presented in the abstract are present and identical to numbers presented in the main manuscript text.

Author response: The numbers in the abstract has been updated.

* In the last sentence of the Abstract Methods and Findings section, please describe the main limitation(s) of the study's methodology.

Author response: The following sentence has now been added to the Abstract (page 4) to highlight the main limitation of our study:

“We emphasise however that, whilst leveraging large-scale genetic association data available provides us with a unique opportunity to evaluate additional indications and mechanisms of PCSK9 inhibition, our estimates are based on a lifelong exposure which makes direct comparisons with trial results challenging.”

At this stage, we ask that you include a short, non-technical Author Summary of your research to make findings accessible to a wide audience that includes both scientists and non-scientists. The Author Summary should immediately follow the Abstract in your revised manuscript. This text is subject to editorial change and should be distinct from the scientific abstract. Please see our author guidelines for more information: <https://journals.plos.org/plosmedicine/s/revising-your-manuscript#loc-author-summary>

Author response: The Author Summary has been added to page 5 as requested.

Please ensure that the study is reported according to the STROBE-MR guideline, and include the completed STROBE-MR checklist as Supporting Information. Please add the following statement, or similar, to the Methods: "This study is reported as per the Strengthening the Reporting of Observational Studies in Epidemiology (STROBE) guideline, specific for mendelian randomization (S1 Checklist)." The STROBE-MR guideline can be found here: <https://www.strobe-mr.org/> When completing the checklist, please use section and paragraph numbers, rather than page numbers.

Author response: The STROBE-MR checklist has been added as S1 Checklist in the supporting documents. Relevant statement has been added to the Methods section (page 8):

“This study is reported as per the Strengthening the Reporting of Observational Studies in Epidemiology (STROBE) guideline, specific for Mendelian randomization [31] (**S1 Checklist**).”

Please delete the competing interests information from references #11, #27, and #66.

Author response: The information has been removed as requested.

**Comments from the reviewers:**

**Reviewer #1:**

The authors present an intriguing drug target MR study to evaluate the benefit of cholesterol lowering drugs to reduce the risk of prostate cancer. Overall, the study is well-conducted, and the analysis is largely convincing. I have a few comments to improve the presentation and suggestions for additional analysis.

1. Data sources: It would be fantastic if the authors could update the genetic associations with LDL-c to use the latest GLGC release instead of the 2013 one.

Author response: Thanks for the suggestion. We have replaced the genetic instruments for exposures with the new GLGC GWAS meta-analysis on LDL cholesterol conducted on European population (Graham et al. 2021 Nature). Relevant information has been updated in the Methods (page 9) and Results (page 15) sections.

2. Data sources: cis-pQTLs genetic associations were estimate in the Icelandic population, yet clumping was performed using a European ancestry panel. How transferable is the European ancestry to the Icelandic ancestry? Could the authors find a better matching reference panel for LD estimation?

Author response: Thank you for this suggestion. The Icelandic plasma proteome GWAS by deCODE genetics provides by far the largest full summary-level genetic association data on the levels of plasma PCSK9 protein. Although the GWAS was not based on central European population, we believe the pQTLs analysis provides highly valuable evidence supporting the results from the primary MR analyses. To demonstrate this, we replicated the PCSK9 pQTL MR analysis using conditionally independent cis-pQTL presented in the original deCODE plasma proteomics paper (Ferkingstad et al. 2021 Nature Genetics). Repeating the MR analysis using these instruments on overall PrCa risk provided a very similar magnitude of effect (OR=0.92 per 1 SD reduction in plasma PCSK9 levels, 95% CI=0.85 to 0.999, *p*=0.048) compared with the results we originally reported in the manuscript.

This analysis has now been added to the manuscript in Methods (page 11) and Results (page 16).

3. Co-localization between eQTL and total prostate cancer risk: Which genetic correlation matrix was used for the calculation of eCaviar?

Author response: The genetic correlation matrix generated using the European individuals from the 1000 genomes reference panel was used for this calculation. This has been added to the Methods section at page 13.

4. Co-localization between eQTL and total prostate cancer risk: Could the authors complement the eCaviar results with the more commonly used colocalisation package coloc or coloc-susie? Looking at the regional Manhattan plot there is only one causal variant in the region, so even standard colocalisation should be able to pick up the signal.

Author response: Thank you for the comment. Based on the reviewer’s suggestion, we have now conducted additional colocalization analysis using coloc R package. The posterior probability (H4) of having a shared SNP between the *PCSK9* liver gene expression and prostate cancer data is 81.3%, whereas the H4 for shared SNP between *HMGCR* or *NPC1L1* liver expression and prostate cancer are below 0.5%.

Relevant information has been added to the Methods (page 13) and Results (page 16-17) sections.

5. Methods: Lipid lowering drugs targets and risk factors of prostate cancer: Would it be possible to use only male-specific summary-level associations for the risk factors (BMI, Lipoprotein (a)) investigated?

Author response: We have added additional replication analysis using the summary-level associations for lipoprotein (a) and body mass index with the largest male-specific GWAS available, including:

a) Pulit et al. GWAS meta-analysis on BMI (n= 374,756 men) when using GLGC variants as the genetic instruments for drug targets,
b) Locke et al. GWAS on BMI (n= 152,893 men) when using UKB male-stratified genetic variants as the genetic instruments for drug targets,
c) The Neale lab GWAS on inverse rank normalised levels of Lp(a) in UKB male participants (n= 167,020 men) (<http://www.nealelab.is/uk-biobank/>).

Relevant information has been added accordingly in the Methods section (page 13) and Results section (page 17-19). Full results can be found in S13-15 Tables.

6. Results: Lipid lowering drug targets and risk factors of prostate cancer
This is an extremely interesting section, yet the author could go one step further. The current analysis only estimates the arrow from Exposure to Mediator in Figure 4. The quantity of interest would be the percentage of effect that is mediated via the prostate cancer risk factors. Could the authors please include a summary-level mediation MR analysis (1) as well or are there too few instruments to obtain reliable estimates?

Author response: Thanks for the question. In mediation & multivariable MR analyses, it is essential to have one or more SNP(s) shared between the genetic variants associated with the exposure and the mediator, which indicate they have a shared effect on the outcome. In the male stratified Lp(a) GWAS on UK Biobank participants, we did not identify any valid instruments around the *PCSK9* gene region, therefore the reviewer is correct that we cannot reliably conduct a mediation MR analysis to estimate the proportion of effect from PCSK9 mediation by Lp(a).

7. Discussion: The authors state: "The lack of evidence for an association between PCSK9 inhibition and the risk of advanced prostate cancer suggest circulating PCSK9 is less likely to be promoting the progression of the disease." Could the authors please perform a power calculation to complement this statement to demonstrate that the lack of evidence is not due to a lack of power.

Author response: We have conducted a post-hoc power calculation using the mRnd web tool (<https://shiny.cnsgenomics.com/mRnd/>):

| Exposure | Outcome | N | OR | K | $R_{xz}^{2}$ | Power |
| --- | --- | --- | --- | --- | --- | --- |
| LDLc mediated by PCSK9 | Overall PrCa | 140,306 | 0.84 | 0.564 | 0.014 | 0.97 |
| LDLc mediated by PCSK9 | Advanced PrCa | 73,475 | 0.85 | 0.206 | 0.014 | 0.52 |
| LDLc mediated by PCSK9 | Early onset PrCa | 51,244 | 0.70 | 0.136 | 0.014 | 0.82 |

where N is the sample size of the outcome GWAS, OR is the odds ratio of having the outcome per 1 SD decrease in LDLc mediated by PCSK9, K is the proportion of cases in the outcome, and $R_{xz}^{2}$ is the proportion of variance explained for the association between the SNP (Z) and the exposure variable (X).

As shown in the table above, the post-hoc power for detecting an odds ratio of 0.85 of having advanced PrCa is relatively low (0.52) compared with the two other analyses, suggesting the lack of evidence could potentially be due to the lack of power. However, post-hoc power calculation uses the odds ratio generated by MR and is potentially biased so we will not include the power statistics into the paper.

We have therefore removed this sentence mentioned by the reviewer. Also, we have added a paragraph discussing this in the Discussion (page 21):

“MR analyses provided a similar central effect estimate for genetically proxied HMGCR estimates and prostate cancer as those found for PCSK9 inhibition, although with wider corresponding confidence intervals resulting in weaker evidence of an effect. Furthermore, as was the case for NPC1L1, there was also weak evidence of a shared causal variant based on colocalization analyses. This could be due to the lack of power provided by the prostate cancer GWAS used in this study, meaning that investigations should be repeated in the future once findings from larger prostate cancer case-control GWAS are available.”

And in the limitations in the Discussion (page 23):

“Moreover, the lack of evidence for an association between PCSK9 inhibition and the risk of advanced prostate cancer, as well as comparatively wider confidence intervals for associations between HMGCR, NPC1L1 and prostate cancer (compared to genetically predicted PCSK9 inhibition) could potentially be due to lack of power. These should be further explored when larger datasets are available.”

8. General: A recent two-sample MR study has shown a potential causal role for Lipoprotein (a), yet very little evidence for LDL-c (2). Could the authors repeat their drug-target MR analysis using genetic associations with Lipoprotein (a) instead of genetic associations with LDL-c?

Author response: Thanks for the question. We agree that it would be interesting to see the effects from genetically proxied drug effects mediated by Lp(a) on prostate cancer. However, this analysis was not possible to undertake as mentioned in response to comment #6, given that no valid genetic instruments were found in the *PCSK9* gene region from the Lp(a) GWAS. This should therefore be explored in the future when larger GWAS on Lp(a) is available.

We have included this in the Discussion section (page 23):

“Fifthly, multivariable MR cannot be applied to explore the mediatory role of Lp(a) due to the lack of genome-wide significant genetic variants in *PCSK9* gene region from the UK Biobank GWAS on Lp(a). It is worth further investigation when a larger GWAS on Lp(a) is available in the future.”

Minor:
-       "Contrasting the genetically proxied associations between lipid lowering drugs targets and risk factors of prostate cancer" while in the Results it is "Contrasting the genetically proxied associations between lipid lowering drug targets and risk factors of prostate cancer". It would be good to harmonize to "drug targets".

Author response: Thanks for pointing this out. We have now harmonized them to “drug targets”.


References

1.       D. Gill et al., Int J Obes (Lond). 45, 1428-1438 (2021).

2.       A. Ioannidou et al., PLOS Medicine. 19, e1003859- (2022).

**Reviewer #2:**

The authors conducted a drug-target Mendelian randomisation analysis (MR) to examine the potential effect of targeting PCSK9, HMGCR, NPC1L1, and LDLR for LDL-C reduction on the risk of prostate cancer. The idea is scientifically and clinically sound and robust. The finding does not directly impact clinical practice but should interest readers of a clinical journal. However, the novelty is weak as follows.

A manuscript with the same idea has been available as follows: Sun et al., Associations of genetically proxied inhibition of HMG-CoA reductase, NPC1L1, and PCSK9 with breast cancer and prostate cancer, Breast Cancer Res, 2022 Feb. doi: 10.1186/s13058-022-01508-0. Sun et al. conducted MR similar to the primary analysis of this submitted manuscript. The primary data sources were the same: quantitative genetic results from the GLGC and the PRACTICAL consortium.

Sun et al. reported an odds ratio of 0.81 (95% CI 0.73-0.90) per 1 standard deviation lower LDL-c predicted with the genetic instrument specifically for PCSK9-related loci. The authors of this submitted manuscript reported 0.84 (0.74-0.96). The same non-significant results were observed in both studies for the other sets of gene variants related to LDL-c. Two studies took different ways to select loci within and near each of the PCSK9 gene and the others. Other methodological differences were present. The current study evaluated UK BioBank data, too, to examine male-specific genetic effects and support reproducibility. Mechanistic analyses and results are available, for example, with the foci on PCSK9 expression and Lp(a). Specific strengths were terrific compared to the study by Sun et al.

However, the authors neither documented nor discussed the limited novelty appropriately, overstating the novelty. The authors should cite the paper by Sun et al. several times in this manuscript. The introduction section should indicate the availability and implication of the study by Sun et al. Then, the authors should justify why this study is essential for the clinical audience, given the previous effort by Sun et al. In the discussion section, the authors should document what additional evidence they could provide above and beyond that from Sun et al.

Author response: Thank you for bringing the Sun *et al* study to our attention*.* As the reviewer mentioned, our study has several advantages over the Sun et al. study, including:

a) Our study adopted a triangulation approach to support the primary two-sample MR analysis using genetic instruments from molecular datasets, including *PCSK9* liver gene expression eQTL and plasma pQTL. We also estimated the probability of colocalization between genetic variants associated with *PCSK9* liver gene expression and PrCa using two complementary approaches to MR (*coloc* and eCAVIAR).
b) In this revision, we identified a larger set of genetic instruments for drug targets inhibition and LDL-c identified from the latest GLGC GWAS meta-analysis on LDL-c (Graham et al. 2021 Nature Genetics), whereas the Sun et al. study extracted their instruments from an older version of GWAS published in 2013.
c) Our study included LDLR and overall LDL-c as exposure, helping to infer whether the association between lipid lowering drugs and prostate cancer is mediated through on-target effects on LDL-c.
d) Our MR analysis using the weakly correlated genetic variants was adjusted for the correlation between those variants using LD matrix, whereas the Sun et al. study did not adjust for the correlation between SNPs.
e) Our study included replication analysis using male stratified GWAS datasets.
f) Our study looked at the effects from genetically proxied inhibition of PCSK9 on early onset and advanced PrCa, whereas the Sun et al. study only examined the effects on overall PrCa risk.
g) Our study explored potential mediators in the association between genetically proxied inhibition of PCSK9 and PrCa risk, providing a hypothesis that the effects is mediated by the levels of Lp(a).

As suggested by the reviewer, we have now cited the Sun *et al.* study (citation #12) in the Introduction (page 6). We have also added a comparison between the Sun *et al.* study with our study in the Discussion section (page 21) which discusses the bullet points raised above.

Minor comments:

The introduction should clarify what MR evidence was available before this study. The publications of #5 and #27, for example, seem to be the prior MR partly promising LDL-C lowering for lowering the risk of prostate cancer. The introduction should clarify what evidence was available from MR or non-genetic observational studies. Otherwise, readers would gain impression that the authors are hiding prior MR evidence about lipids and prostate cancer.

Author response: Thank you for this comment. Publications #5 (Bull *et al.,* 2015) and #27 (Ioannidou *et al.*, 2022) both investigated the associations between genetic instruments for overall LDL-c and PrCa risk. Bull *et al.* found weak evidence for the association between higher LDL-c and overall PrCa risk (PR=1.24, 95% CI=0.90 to 1.69, P=0.18, per genetically instrumented SD increase in LDL-c). In addition, although Ioannidou *et al.* (using genetic variants identified from UK Biobank LDL-c GWAS) did identify an association between LDL-c and PrCa risk (OR=1.088, 95% CI = 1.010 to 1.162; P = 0.016, per SD increase in genetically predicted LDL), the result was not supported by pleiotropic robust methods. Further investigation using multivariable MR found no evidence for the association when adjusting for other lipid traits. Thus, we cannot present these two studies as evidence supporting the association between LDL-c lowering and a lower risk of PrCa.

We have now added the Ioannidou *et al.* paper into the introduction and modified the sentence to mention classic observational studies and MR evidence on the associations between LCL-c and prostate cancer (page 6) separately:

“Conversely, classic observational studies [4, 5] and MR analyses [6-8] have typically found limited evidence of an association between the levels of LDL-c and overall risk of prostate cancer, although some have reported that LDL-c lowering medications may reduce the risk of prostate cancer incidence [6, 9-12].”

The authors may better provide Miami plots for GWAS results for LDL-c and prostate cancer for each genetic region of interest (PCSK9, HMGCR, etc.). Figure 1 visually indicates the genetic correlations between genetic effects on LDL-C and those on prostate cancer. It would be nice to see the same figures for the other genes in the supplementary materials: those may not visually indicate any genetic correlations or one promising for HMGCR.

Author response: Thank you for this suggestion. We have added two additional locus zoom plots showing the *HMGCR* and *NPC1L1* locus from liver derived gene expression and prostate cancer GWAS as supporting files S2 Fig and S3 Fig. In addition, we have also added the locus zoom comparing the LDL cholesterol and PrCa GWAS at *PCSK9*, *HMGCR* and *NPC1L1* locus as supporting files S4 Fig, S5 Fig and S6 Fig respectively.

The authors may better quantify genetic correlations without selecting specific loci for each gene with user-defined criteria. The authors may do LD-score regression as secondary analysis, subsetting the genetic data to a particular genetic region.

Author response: Thank you for the comment. We conducted LD score regression on LDL-c and PrCa GWAS and identified evidence for association between the two traits using genome-wide variants (correlation coefficient=0.062, 95% CI=0.009 to 0.115, P=0.022). We have conducted additional local genetic correlation analysis using LAVA (v0.1.0) (<https://www.nature.com/articles/s41588-022-01017-y>) to compare the genetic correlation between the levels of LDL-c, PCSK9 plasma protein and the risk of prostate cancer. The genetic data for all phenotypes were subset to a region up- and down-stream 1Mb around the lead SNP (rs553741) in the PrCa GWAS at the *PCSK9* locus. Liver specific *PCSK9* gene expression GWAS dataset was not analysed because LAVA detected negative variance estimate for it. Both LDL-c (correlation coefficient rho=1) and PCSK9 protein (rho= 0.78) datasets are highly correlated with PrCa data at the 2Mb genetic region with 95% confidence interval of the correlation coefficient rho containing 1.

The analysis on LDL-c and PrCa have been added to Methods (page 13), Results (page 17) and supporting file S12 Table.

Discussion:

The authors should discuss the clinical pharmacological effect of lowering LDL-c or Lp(a) was comparable with the estimate from MR. The authors have documented the potential lack of the comparability as a limitation. However, they can collectively and quantitatively discuss it based on their estimate and published information.

Author response: Thanks for the suggestion. Findings from the FOURIER trial reported that PCSK9 inhibitors lower LDL-c by a median of 0.78 mmol/L, which suggests that the putative additional indication towards lower PrCa risk based on our genetically predicted estimates (i.e., a 15% reduction in odds of PrCa per 0.87 mmol/L lower LDL-c) should in theory lead to a clinically meaningful benefit. That said, this comparison is challenging to make given that the genetic variants used as instrumental variables in our study capture a lifelong effect, whereas PCSK9 inhibitors prescribed during the FOURIER trial (where the median duration of follow-up was 2.2 years) lead to a temporary reduction in LDL-c levels. In addition, our analysis is based on non-clinical samples from the general population, whereas the FOURIER trial recruited those with a prior cardiovascular disease history and at high risk of recurrence and are also prone to factors such as non-adherence and contamination. It should be emphasised that our study investigates target-mediated effects of medication instead of the actual effect of medication themselves. Therefore, the findings should not be interpreted as encouraging the repurposing of PCSK9 inhibitors for prostate cancer. The beneficial role of PCSK9 inhibition in reducing the risk of prostate cancer should be investigated in further studies such as randomised controlled trials.

Other minor comments are reserved at this moment.

**Reviewer #3:**

The authors present an analysis of strong association of the genetically proxied inhibition of PCSK9 with a lower risk of overall and early onset prostate cancer, potentially through a mechanism involving the lowering of Lp(a) levels. The authors explored a very interesting and clinically relevant question utilizing previously collected genetic sequencing and phenotype data on a cohort of men in UK Biobank study.

Major Comments:

1. Authors should provide a table on Summary of Demographics and Clinical Characteristics of the study cohort

Author response: Thanks for your comments to help refine this work. To provide a better picture of data involved in our study, we have added a new supplementary table (S1 Table) to present the relevant information gathered from each of the GWAS studies as much as possible.

2. Authors should describe in methods: how they defined the advanced prostate cancer and controls in specific groups (early onset and advanced PCa group) and if the case and controls are matched (eg. age, serum PSA, Family history, clinical follow-up time)? Was the analysis adjusted to significant confounding variables?

Author response: To achieve the maximum possible statistical power in this study we used data provided by the PRACTICAL consortium who meta-analysed many different cohorts with PrCa cases and non-PrCa individuals as controls. These data have been widely analysed in the MR field to date including several publications in PLOS Medicine. However, by using these data it was not possible for us in this study to match controls for confounding variables mentioned by the reviewer. This has been added to the Methods (page 11).

3. Authors should provide functional evidence of how the variants affects the gene and regulatory pathway for lipoprotein metabolism in context of PCa.

Author response: We have now added the consequences of all genetic variants used as instrumental variables for PCSK9 to Tables S5 based on Ensembl annotations. Regulatory pathway data for all targets analysed in this study was queried using the STRINGdb database based on experimentally determined data (S1 Fig) which is now referenced on page 11.

Minor Comments:

1. How the association for genetically proxied inhibition of PCSK9 stands, when the advanced PCa group is split in subgroups (eg. metastasis group, high Gleason grade group etc.). The results may be better refined since advanced PCa considered in the study is heterogenous.

Author response: Thanks for your comments. We agree with the reviewer that it would be highly interesting to examine the association between genetically proxied inhibition of PCSK9 and PrCa subgroups and stratified phenotypes. However, we are limited by the data available provided by the PRACTICAL consortium in this study. Their current stratified PrCa GWAS (other than early-onset and advanced PrCa already analysed, where non-PrCa individuals were used as controls) were entirely based on PrCa cases without non-cancer controls. An MR analysis using such datasets may induce index event bias (IEB), leading to false negative results or even spurious inverse associations with an outcome depending on the strength of the association between traits colliding on another trait and their own relationships with the outcome.

For example, attempting to analyse the high Gleason grade group from PRACTICAL provides weak evidence of an effect (Beta=0.04, 95%CI= -0.12 to 0.06, *p*=0.487), although this null finding could be biased due to the fact that GWAS samples were stratified based on PrCa cases only (i.e., as opposed to comparison with non-PrCa participants).

Therefore, we have added a few sentences in the discussion advocating the future analysis of these PrCa subgroups once well-powered and appropriate GWAS samples are available (page 23-24):

“Furthermore, we did not explore the genetically proxied association between drug target inhibition and other stratified prostate cancer phenotypes, such as Gleason score and prostate cancer aggressiveness. Although the PRACTICAL consortium has published GWASs on these phenotypes, these GWASs are conducted among prostate cancer cases without the inclusion of non-cancer controls. MR analysis using such datasets may induce index event bias [79], leading to false negative results or even spurious inverse associations.”

Lastly, whilst statistical methods have been developed to detect and adjust for IEB, such as “Slope-Hunter” (<https://www.nature.com/articles/s41467-022-28119-9>) and the "Dudbrige" method (<https://www.nature.com/articles/s41467-019-09381-w>), both methods require estimation of the slope of SNP-prognosis/SNP-incidence effects across large numbers of independent SNPs. This is therefore not possible in our study due to the limited number of cis-acting variants to proxy the inhibition of PCSK9.

2. Since the treatment regimen and age can affect the levels of hormones and lipids in circulation, authors may want to comment on these aspects and how they incorporated this variability in the present study

Author responses: We have now added some discussion to highlight the importance of factors such as ongoing treatments and the age of participants and how these may impact findings in MR studies such as ours (page 24):

“Similarly, leveraging large-scale summary-level data from PRACTICAL meant that we were unable to evaluate the time-varying effects of PCSK9 inhibition on risk of PrCa at separate stages in the lifecourse, or in population subgroups undergoing different treatment regimens. These are therefore important areas of future research to investigate either in a clinical trial setting, or when large-scale genetic association datasets become accessible.”

3. Authors may include analysis on biochemical recurrence, if clinical follow up on patients is available.

Author responses: As discussed above, our study is based on summary level datasets which were required to aggregate a large number of PrCa cases with genotype data. Therefore, clinical follow up on individuals is not available in this work and so this point has been mentioned in the limitations (page 23-24):

“Furthermore, we did not explore the genetically proxied association between drug target inhibition and other stratified prostate cancer phenotypes, such as Gleason score, cancer aggressiveness or recurrence. Although the PRACTICAL consortium has published GWASs on some of these phenotypes, these GWASs are conducted among prostate cancer cases without the inclusion of non-cancer controls. MR analysis using such datasets may induce index event bias [79], leading to false negative results or even spurious inverse associations. These could be investigated by following up participants of randomized controlled trials for PCSK9 inhibitors.”
