# Supplementary material for: Association between genetically proxied PCSK9 inhibition and prostate cancer risk: A Mendelian randomisation study
Source: PLoS Med. 2023 Jan 3;20(1):e1003988. doi: 10.1371/journal.pmed.1003988 (PMC9810198; doi:10.1371/journal.pmed.1003988)
Supplement: Attachment — Submitted filename: pcak9_prostate_cancer_Response to reviewers comments_R3.docx [file pmed.1003988.s027.docx]

**Manuscript ID: PMEDICINE-D-22-01177R3**

**Response to Reviews’ Comments**

Dear PLOS Medicine editorial team and reviewers,

Thank you for your further comments regarding our manuscript titled “Association between genetically proxied PCSK9 inhibition and prostate cancer risk: a Mendelian randomization study”. We would also like to thank the editor and reviewers for reviewing our manuscript again. The manuscript, especially the introduction section, has been updated according to the comments from reviewer #2.

We can confirm that this manuscript has not been published and is not under consideration for publication elsewhere. For the purpose of Open Access, the author has applied a CC BY public copyright licence to any Author Accepted Manuscript version arising from this submission.

We look forward to receiving the proof of the manuscript for publication.

Yours faithfully,

Si Fang

on behalf of authors

**Requests from Editors:**

The Data Availability Statement (DAS) requires revision. If data are freely available upon request, please state the owner of the data set and contact information for data requests (web or email address). Note that a study author cannot be the contact person for the data.

Author response: The summary statistics for male-specific GWAS on LDL cholesterol will be uploaded to IEU OpenGWAS portal, allowing public access without restrictions. This has been added to the DAS.

The ‘Author Contributions’, ‘Funding’, and ‘Conflicts of Interest’ sections can all be removed from the main manuscript, but it is essential that this information is all captured via your responses to the submission form – when submitting your revision, please ensure all information is included in your answers to the relevant questions.

Author response: These three sections has been removed from the main manuscript.

Please include continuous line numbering throughout your manuscript.

Author response: Continuous line numbers have been added to the manuscript.

Please ensure that all numbers presented in the abstract are present and identical to numbers presented in the main manuscript text.

Author response: We have checked the numbers and they are now consistent with results reported in the manuscript.

To help us extend the reach of your research, please provide any Twitter handle(s) that would be appropriate to tag, including your own, your coauthors’, your institution, funder, or lab. Please respond to this email with any handles you wish to be included when we tweet this paper.

**Comments from Reviewers:**

**Reviewer #1:** The authors have addressed all of my comments.

Author response: Thank you for reviewing our manuscript again!

**Reviewer #2:** The authors revised the manuscript reasonably well. The Reviewer has come up with minor comments, but the revision of the introduction should be made clearly accordingly.

Abstract:

Methods and findings: The data use should be more explicit. The authors should clarify whether they meant the use of publically available summary statistics or new in-house or collaborative analyses. "SNPs were used" and "Association estimates were obtained" are unclear. In recent MR literature, using summary statistics only seems to be common. If the authors did otherwise, e.g. sex-specific GWAS for lipids, they may want to clarify it. The authors may wish to clarify it in Figure 1, too.

Author response: Thank you for the comment. “Summary-level data” has been added to the abstract to clarify the instrument selection process as well as mentioning that ‘two-sample MR’ was applied in this study. The detailed description of datasets used in this work (e.g. sex-stratified GWAS) can be found in the Data Availability Statement (DAS).

The introduction section on page 6 requires some re-organisation. The authors explain MR analysis in the second paragraph but introduce the existing evidence based on MR in the first paragraph.

Page 6 should explicitly include the information on previous MR analyses that linked lipid-lowering loci of the specific genes to prostate cancer risk.

In the second paragraph, the authors should cite the ref #12, present the previous results from the most relevant MR analysis, and explain why this study was necessary over and above what Ref 12 showed. As previously commented, the MR analysis conducted by Sun et al. (Ref 12) has already highlighted the idea of the drug-targetted MR for prostate cancer research. The authors should not introduce the approach as if they did it first in the context of lipid-lowering drugs and prostate cancer prevention. It would be expected to see a sentence starting with "Sun et al. did XXX. They reported XXX."

Author response: The introduction section has been edited according to the suggestions.

On Page 8, "Our work involves" should be in the past tense.

Author response: “Our work involves” has been edited to the past tense.

Figure 2B is better to put in the supplementary material. Compared to Figure 2A, 2B is not necessary, just supporting the robustness of the PCSK9 result.

Author response: Figure 2B has been moved to supplementary material (new S2 Fig).

Figure 2A should have a greater information on the left label. The gene information may better be supplemented with the number of SNPs and F statistics, for example.

The legend of Figure 2 should include the exposure unit: e.g. genetically estimated 1-SD increase in LDL-cholesterol in the GLGC.

Author response: Relevant information has been added to the legend for Figure 2 (which is now Figure 2A after Figure 2B was moved to supplementary as above):

“**Fig 2. Results from Mendelian randomization analyses to estimate the effect of lipid lowering therapies and risk factors on overall prostate cancer risk.** Effect estimates are odds ratios for prostate cancer per 1 standard deviation (SD) reduction in LDL cholesterol (LDL-c) proxied using genetic instruments identified from the Global Lipids Genetics Consortium (GLGC). A total of 28, 4, 13, 26 and 424 genetic instruments were selected to predict the effects of PCSK9 inhibitors, NPC1L1 inhibitors, HMGCR inhibitors, the inhibition of LDLR and the reduction of LDL cholesterol, respectively. F-statistics for the exposures examined ranges from 221.5 to 629.7.”

Figure 4 legend should say "Effect estimates were in..." as the exposure unit the authors used in their analysis.

Author response: The legend for Figure 4 has been modified accordingly.

**Reviewer #3:** The authors have very well addressed my comments and critiques.

Author response: Thank you for reviewing our manuscript again!
